# Locating multiple diffusion sources in time varying networks from sparse observations

**DOI:** 10.1038/s41598-018-20033-9

**Published:** 2018-02-08

**Authors:** Zhao-Long Hu, Zhesi Shen, Shinan Cao, Boris Podobnik, Huijie Yang, Wen-Xu Wang, Ying-Cheng Lai

**Affiliations:** 1 College of Mathematics, Physics and Information Engineering, Zhejiang Normal University, Jinhua, 321004 Zhejiang, China; 2 School of Systems Science, Beijing Normal University, Beijing, 100875 China; 3 School of Finance, University of International Business and Economics, Beijing, 100029 P. R. China; 4 Center for Polymer Studies Boston University, Boston Massachusetts, 02215 USA; 5 Faculty of Civil Engineering, University of Rijeka, 51000 Rijeka, Croatia; 6 Business School, University of Shanghai for Science and Technology, Shanghai, 200093 China; 7 School of Electrical, Computer and Energy Engineering, Arizona State University, Tempe, Arizona 85287 USA; 8 Department of Physics, Arizona State University, Tempe, Arizona 85287 USA

## Abstract

Data based source localization in complex networks has a broad range of applications. Despite recent progress, locating multiple diffusion sources in time varying networks remains to be an outstanding problem. Bridging structural observability and sparse signal reconstruction theories, we develop a general framework to locate diffusion sources in time varying networks based solely on sparse data from a small set of messenger nodes. A general finding is that large degree nodes produce more valuable information than small degree nodes, a result that contrasts that for static networks. Choosing large degree nodes as the messengers, we find that sparse observations from a few such nodes are often sufficient for any number of diffusion sources to be located for a variety of model and empirical networks. Counterintuitively, sources in more rapidly varying networks can be identified more readily with fewer required messenger nodes.

## Introduction

Diffusion and propagation processes taking place in complex networks are ubiquitous in natural and in technological systems^1,2^, Examples of those processes include air or water pollution diffusion^3,4^, disease or epidemic spreading in the human society^5,6^, virus invasion in computer and mobile phone networks^7,8^, behavior propagation in online social networks^9^. Once a negative diffusion or propagation emerges, it is imperative to locate its sources quickly and precisely to enable timely and appropriate control strategies to prevent and/or inhibit the spreading process. A number of methods have been proposed and tested recently to address the source localization problem of propagation processes in complex networks, which include those based on the maximum likelihood estimation^10^, dynamic message passing^11^, belief propagation^12^, hidden geometry of contagion^13^, and inverse spreading^14,15^, A related problem of practical significance is to identify super spreaders for effective control of spreading^16,17^, However, most existing approaches are specifically for static networks. In the real world time varying networks are ubiquitous, such as frequently changed social contacts via meetings, emails, phone and online softwares^18–21^, Recently, a source detection framework was proposed on complex networks from one snapshot observation of the entire network and demonstrated for an empirical temporal network of sexual contacts^22^.

**Figure 1:**
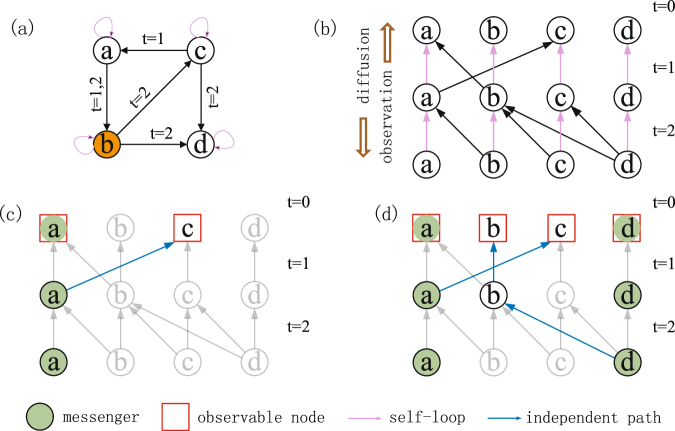
Illustration of proposed framework to locate multiple diffusion sources in time varying networks. (**a**) A simple directed time varying network where the numbers associated with the edges denote the activation time, where node *b* is the source and self loops are specified by the nonzero diagonal elements of matrix *A*. (**b**) Static mapping of the network in (a), where each layer corresponds to an activation time. The diffusion direction is from top to bottom (from *t* = 0 to *t* = 2), while observations occur in the opposite direction. (**c**) An independent path from observing messenger node *a*, where the observable range is *N*_OR_({*a*}) = 2. (**d**) Two independent paths from observing nodes *a* and *d*. The network is fully observable (*N*_OR_({*a*,*d*}) = 4) and sources are fully locatable.

Those works focus primarily on source localization for propagation processes. However, source localization for diffusion processes is rarely studied. Here we concentrate on diffusion processes, as they constitute a good approximation for different types of dynamical processes (e.g., synchronization and other nonlinear processes amenable of linearization)^2^. Very recently, considering multiple sources may exist (e.g., air or water pollution, rumors), a general framework that locating of multiple sources in static diffusion processes is presented^23^. To develop effective frameworks to locate sources in time varying networks is an outstanding problem in network science and engineering. The essential difference between diffusion on a time varying network and on a static network is illustrated in Fig. 1. Specifically, in Fig. 1(a), due to the various time intervals in which different edges are activated, a spreading process starting at node *b* cannot reach node *a* in any time. In contrast, for a static network with the same structure as shown in Fig. 1(a), the spreading process can reach all nodes in the network. To our knowledge, there has been no solution to the problem of locating multiple diffusion sources associated with general dynamical processes on arbitrary time varying networks from local observations^24^. The purpose of this paper is to provide an optimal solution. In particular, exploiting a combination of the structural observability and sparse signal reconstruction theories, we develop a general source localization framework that is applicable to arbitrarily time varying networks with any number of sources. We demonstrate that sparse data from a small set of messenger nodes are capable of identifying multiple diffusion sources accurately and efficiently, even in the absence of detailed information about the network structure such as link weights and the presence of measurement noise. The framework is established analytically and validated through extensive numerical tests of model and empirical networks.

## Results

### Framework of locating multiple sources on time-varying networks

A time-varying network with *N* nodes is generally defined by a node set *V* = {*v*_1_, *v*_2_, ..., *v*_*N*_} with a set *E* of time varying edges, where (*v*_*i*_, *v*_*j*_, *w*_*ji*_, *t*) ∈ *E* denotes a directed edge pointing from nodes *v*_*i*_ to *v*_*j*_ with link weight *w*_*ji*_ at activation time *t*. In this paper, we consider the following class of discrete-time, diffusion processes on such time varying networks:

1 $${x}_{i}(t+\mathrm{1)}={x}_{i}(t)+\beta \sum _{j=1}^{N}[{w}_{ij}(t+\mathrm{1)}{x}_{j}(t)-{w}_{ji}(t+\mathrm{1)}{x}_{i}(t)],$$

where *x*_*i*_(*t*) is the state of node *i* at time *t* capturing the fraction of infected individuals, the concentration of water or air pollutant and etc., at place *i*. *β* is the constant diffusion coefficient, and *w*_*ij*_(*t*) is the link weight at time *t*, where self loops are a result of the diffusion process^2^. For an undirected network, we have *w*_*ij*_(*t*) = *w*_*ji*_(*t*). (Diffusion dynamics in continuous time can be treated similarly - see Sec. S1 in Supplemental Information (SI)). The nodes from which observations are made are the *messenger nodes*. When the outputs from the messenger nodes are taken into account, the system becomes

2 $$(\begin{array}{l}{\bf{x}}(t+\mathrm{1)}=A(t+\mathrm{1)}{\bf{x}}(t),\\ {\bf{y}}(t)=C{\bf{x}}(t),\end{array}$$

where the state vector ${\bf{x}}(t)\in {{\mathbb{R}}}^{N}$ comprises all nodes in the network at time *t* and *A*(*t* + 1) = *I* + *βL*(*t* + 1). In *A*(*t* + 1), $I\in {{\mathbb{R}}}^{N\times N}$ is the identity matrix, *L*(*t*) = *W*(*t*) − *D*(*t*) is the network Laplacian matrix at time *t*, $W(t)\in {{\mathbb{R}}}^{N\times N}$ is the weighted adjacency matrix of elements *w*_*ij*_(*t*), and $D(t)\in {{\mathbb{R}}}^{N\times N}$ is a diagonal matrix of elements *d*_*i*_(*t*) denoting the total out-weight ${\sum }_{j\in {\Gamma }_{i}(t)}{w}_{ji}(t)$ of node *i* with Γ_*i*_(*t*) being the neighboring set of *i* at time *t*. The vector ${\bf{y}}(t)=[{y}_{1}(t);{y}_{2}(t);\cdots ;{y}_{q}(t)]$ represents the *q* measurable outputs from *q* messengers at time *t*, and $C\in {{\mathbb{R}}}^{q\times N}$ is the *output matrix*, where *C*_*ij*_ = 1 if output *y*_*i*_(*t*) is measured from node *j*. The basic difference between source nodes and passive nodes is that, initially (*t* = *t*_0_), the states of the former and latter are nonzero and zero, respectively. Without loss of generality, we set *t*_0_ = 0. Thus, if the initial states of all nodes can be recovered from the measurements of the messenger nodes at a later time (*t* > 0), all sources can be identified. A solution to this problem can be obtained by exploiting the observability condition in canonical control theory. Specifically, we consider instants of time *t* = 0, 1, ..., *T* and rewrite Eq. (2) as

3 $${\bf{Y}}=(\begin{array}{c}{\bf{y}}\mathrm{(0)}\\ {\bf{y}}\mathrm{(1)}\\ \vdots \\ {\bf{y}}(T)\end{array})=(\begin{array}{c}C\\ CA\mathrm{(1)}\\ \vdots \\ CA(T)A(T-\mathrm{1)}\cdots A\mathrm{(1)}\end{array}){\bf{x}}\mathrm{(0)}\equiv O\cdot {\bf{x}}\mathrm{(0).}$$

where ${\bf{Y}}\in {{\mathbb{R}}}^{q(T+\mathrm{1)}}$, ${\bf{x}}\mathrm{(0)}\in {{\mathbb{R}}}^{N}$ is the initial state vector, *q* is the number of messenger nodes, and $O\in {{\mathbb{R}}}^{q(T+\mathrm{1)}\times N}$ is the observability matrix. To be able to accurately locate the diffusion sources, a unique solution of Eq. (3) is needed, given the output vector **Y** from the set of messenger nodes. The classic observability theory stipulates that, if and only if matrix *O* has full rank, i.e., rank(*O*) = *N*, **x**(0) can be fully and uniquely determined.

If we observe only a single node *v*, matrix *O* may not have full rank. As a result, only the initial states of a subset of nodes in **x**(0) can be reconstructed. The number of nodes whose initial states can be reconstructed is rank(*O*), which defines the observable centrality *N*_OR_({*v*}) of *v*, i.e., *N*_OR_({*v*}) = rank(*O*). Analogously, for a given set *Q* of nodes, we have an associated matrix *C* and can obtain rank(*O*), which defines the observable range *N*_OR_(*Q*) of *Q*, i.e., *N*_OR_(*Q*) = rank(*O*). Note that *N*_OR_({*v*}) ≤ *N* and *N*_OR_(*Q*) ≤ *N*. Thus we can define a normalized observable centrality *n*_OR_({*v*}) ≡ *N*_OR_({*v*})/*N* and a normalized observable range *n*_OR_(*Q*) ≡ *N*_OR_(*Q*)/*N*.

Since information about the link weights may not be available, a direct calculation of rank(*O*) is not feasible. A resolution is to analyze the structural observability^25–28^, which is a highly nontrivial task for time varying networks. Our idea is to exploit the independent paths in static mappings of the underlying network^29^, as shown in Fig. 1(b). In particular, a mapping from a time varying network to a static network can be obtained by cloning all nodes into different layers that correspond to different time *t*. If an edge is active at *t* [as shown in Fig. 1(a)], the two nodes at both ends of the edge in the corresponding layers in Fig. 1(b) will be connected. Note that the direction of links in Fig. 1(b) is reversed with respect to the actual direction of diffusion in Fig. 1(a) - a consequence of the duality relation between structural observability and controllability^28^.

Figure 1(c) indicates the quantity *N*_OR_({*a*}) when node *a* is chosen as a messenger node. There is a single independent path, i.e., *a* → *c*, such that *N*_OR_({*a*}) = 2 (one independent path and *a* itself). If *a* and *d* are messengers [Fig. 1(d)], there are two independent paths and *N*_OR_({*a*, *d*}) = 4 (including the two messengers themselves). In this case, the network is fully observable. The key to source localization is thus to identify all independent paths from messenger nodes in the static mappings of the original time varying network. In this paper, to generate a time-varying network, we propose a uniform activation network model in which random activations are imposed on a static network. Specifically, let *z* be the number of times (activations) an edge is active in a time interval, which can be randomly selected from a uniform distribution $U\mathrm{(1},{z}_{\max })$ with ${z}_{\max }$ denoting the maximum number of activations. After *z* is given for each edge, the active time associated with each activation is uniformly chosen from the distribution *U*(1, *T*) under the constraint that a link cannot be activated twice (or more) at one active time.

### Estimate of observable range

For a set *Q* of messenger nodes, *N*_OR_(*Q*) is exactly the number of independent paths plus the number of the messengers, which can be calculated by using the maximum flux algorithm. Here, we provide a theoretical estimate of the number of independent paths. As shown in Fig. 1, since every node has a self-loop, if there exists a link for a certain layer (*t* > 0), there must exist a path starting from the layer to the top layer (*t* = 0), as shown in Fig. 1(d). Moreover, there exists at most one independent path starting from one node in a given layer (*t* > 0). Thus, for a messenger node *v*, the maximum number of independent paths from *v* for all layers is the number of layers in which *v* has a link that points to other nodes. The number is nothing but the number *l*_*v*_ of distinct activations of *v*, where each activation (active time) corresponds to a layer with a link going out from *v* (see Sec. S2 in SI for more details). Thus, since the overlap among independent paths from *v* is negligible, we have *n*_OR_({*v*}) ≈ (*l*_*v*_ + 1)/*N*, based on which the quantity *n*_OR_(*Q*) of node set *Q* can be estimated as

4 $${n}_{{\rm{OR}}}(Q)\approx \sum _{i\in Q}({l}_{i}+\mathrm{1)}/N\mathrm{.}$$

The fraction *p* of messenger nodes is thus *p* = *q*/*N*, where *q* is the number of messengers.

For the uniform activation network model, if the number of distinct activations, *l*_*v*_, cannot be directly measured, we can use the activation times distribution $U\mathrm{(1},{z}_{\max })$ and the active time distribution *U*(1, *T*) to estimate the average number 〈*l*〉 of distinct activations. Specifically, for a node with *k* edges, we denote their activations by *z*^1^, &hellipsis;, *z*^*k*^. The probability of the number of distinct activations being *l* for one node with *z*^1^, ..., *z*^*k*^ is given by (see Sec. S2 in SI)

5 $$\begin{array}{l}P(l|({z}^{1},\cdots ,{z}^{k}))={c}_{1}(\begin{array}{c}T\\ l\end{array})\sum _{j=\,\max ({z}^{1},\ldots ,{z}^{k})}^{l}{(-\mathrm{1)}}^{l-j}(\begin{array}{c}l\\ j\end{array})\prod _{i}^{k}(\begin{array}{c}j\\ {z}^{i}\end{array}),\end{array}$$

where *c*_1_ is a normalization constant satisfying

6 $$\sum _{l=\,\max ({z}^{1},\ldots ,{z}^{k})}^{\min (T,\sum _{i}{z}^{i})}P(l|({z}^{1},\cdots ,{z}^{k}))=1.$$

Therefore, for one node associated with *z*^1^, ..., *z*^*k*^, the average number of distinct activations is

7 $${\langle l\rangle }_{\{{z}^{1},\ldots ,{z}^{k}\}}=\sum _{l=\,\max ({z}^{1},\ldots ,{z}^{k})}^{\min (T,\sum _{i}{z}^{i})}lP[l|({z}^{1},\cdots ,{z}^{k})]\mathrm{.}$$

For a node of degree of *k*, the average number of distinct activations is

8 $$\langle l\rangle ={c}_{2}\sum _{{z}^{1}=1}^{{z}_{\max }}\ldots \sum _{{z}^{k}=1}^{{z}_{\max }}{\langle l\rangle }_{\{{z}^{1},\ldots ,{z}^{k}\}},$$

where ${c}_{2}={({z}_{\max })}^{-k}$. Given 〈*l*〉 for each node, for the entire messenger set *Q*, the normalized observable range can be approximated as

9 $${n}_{{\rm{O}}R}(Q)\approx \sum _{i\in Q}(\langle {l}_{i}\rangle +\mathrm{1)}/N\mathrm{.}$$

### Messenger selection

Considering the cost of allocating messengers for monitoring the state of the whole network, finding a minimum set of messengers through independent paths represents the most efficient way to locate sources. Moreover, the set can be used to characterize the source locatability of the network. The difficulty is that this task is NP-complete^30^. We employ an alternative approach by exploiting a greedy optimization algorithm to maximize the observable range *n*_OR_ through selection of the messenger set (see Sec. S3 in SI). In addition, sub-modularity^31,32^ is exploited to reduce the computational cost and provides guaranteed performance at least (1 − 1/*e*) ≈ 0.63 compared to the global optima.

**Figure 2:**
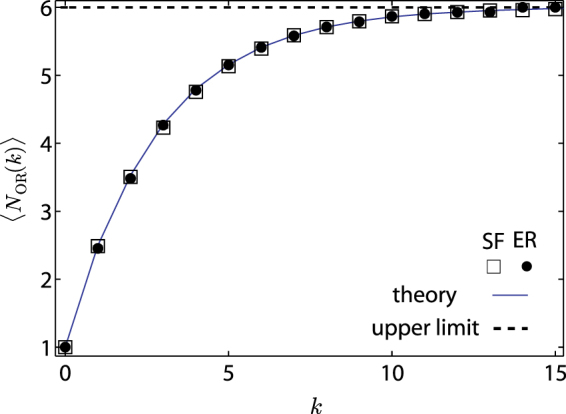
Observable centrality of a single messenger of degree *k* in ER random and SF networks, where the theoretical prediction is from Eq. (9) and numerical results are obtained from averaging over 10,000 independent realizations. The vertical bars indicate the standard error. Observable centrality increases with the degree and approaches its upper limit 〈*N*_OR_〉 = *T* + 1. Other parameters are *N* = 1000, 〈*k*〉 = 6, ${z}_{\max }=2$ and *T* = 5.

We test our framework using model and empirical networks. Figure 2 shows the observable centrality of nodes for Erdös-Rényi (ER)^33^ random and scale-free (SF)^34^ networks. Three features are found, which do not occur for static networks^35^. First, nodes of larger degree *k* have a higher observable centrality *N*_OR_, in sharp contrast to what happens in a static network where both driver and messenger nodes tend to avoid large degree nodes due to their small controllable and observable range. Second, *N*_OR_ gradually approaches the upper limit *T* + 1 as *k* increases. Third, *N*_OR_ is nearly independent of the network structure and depends mainly on *T* and ${z}_{\max }$. The theoretical prediction [Eq. (9)] and numerical results agree well with each other.

**Figure 3:**
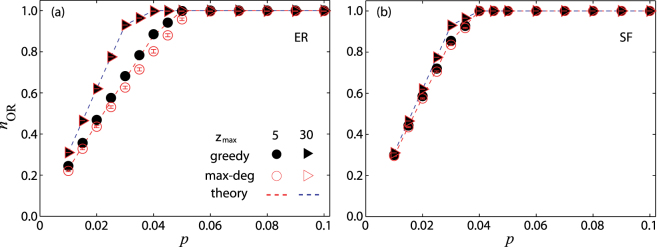
Normalized observable range *n*_OR_ as a function of the fraction *p* of messenger nodes for (**a**) ER and (**b**) SF networks, using the greedy algorithm and max−deg strategy for different values of ${z}_{\max }$, for *T* = 30. The analytical results (dashed curves) from the $\max \,-{\rm{d}}eg$ strategy are from Eq. (9). Network parameters are *N* = 100 and 〈*k*〉 = 6. All results are obtained by averaging over 50 independent realizations and the vertical bars indicate the standard error.

**Figure 4:**
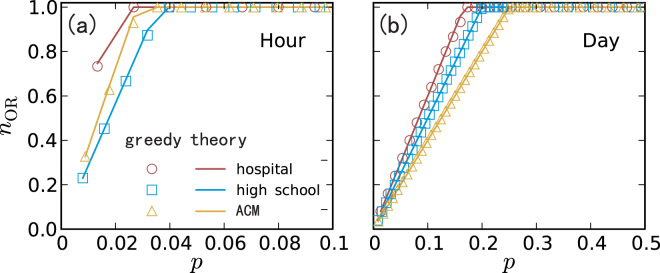
Normalized observable range *n*_OR_ as a function of the fraction *p* of messenger nodes for three empirical networks: Hospital, High School, and ACM. The time windows for (**a**) and (**b**) are one hour and one day, respectively. A greedy algorithm for finding the messenger nodes is used. The theoretical predictions (the solid curves) are from *n*_OR_(*Q*) ≈ ∑_*i*∈*Q*_(*l*_*i*_ + 1)/*N*. Details of the empirical networks and the meaning of the time window can be found in Table S1 and Sec. S4 in SI.

The results in Fig. 2 suggest that large-degree nodes be chosen as the messengers (denoted as the max-deg strategy). To validate this strategy, we compare it with the more elaborative strategy of greedy optimization. As shown in Fig. 3, *n*_OR_ resulting from the max-deg strategy is quite close to that from the greedy strategy, especially for relatively larger values of ${z}_{\max }$. The great advantage of the max-deg strategy is that it is based on *local information only* whereas the greedy strategy requires global information about the network. Another remarkable finding is that a very small fraction *p* of messenger nodes are sufficient to fully locate multiple sources (*n*_OR_ = 1) for both ER and SF networks. We also test our framework using three empirical time varying networks, as shown in Fig. 4. It should be noted that the number of distinct activations *l* of every node is available. We see that a quite small value of *p* can ensure a complete localization of diffusion sources in all the empirical networks. For both model and empirical networks, numerical calculations are in good agreement with theoretical predictions (see Sec. S3 in SI for more details).

A counterintuitive phenomenon is that, in both model and real networks, it is relatively easier to locate diffusion sources in more rapidly changing (more frequently updating) networks as the set of required messenger nodes is smaller (e.g., comparing ${z}_{\max }=5$ with ${z}_{\max }=30$ in Fig. 3 and hour with day in Fig. 4). A heuristic explanation is that more rapid changes in the network structure in fact limit the spreading patterns from sources, facilitating source localization from a relatively smaller number of messenger nodes.

### Actual localization of multiple diffusion sources

We articulate an efficient and robust method to actually locate the sources based on the already identified messenger set. In a realistic situation, the number of sources is much smaller than the network size, so the vector **x**(0) in Eq. ((3)) has many zero elements. The sparsity of **x**(0) can be exploited to greatly reduce the required measurement from messengers by using the compressive sensing (CS) paradigm for sparse signal reconstruction^36,37^, Specifically, Eq. (3) can be solved and accurate reconstruction of **x**(0) can be achieved through solutions of the following convex-optimization problem:

10 $${\rm{\min }}\,\parallel {\bf{x}}\mathrm{(0)}{\parallel }_{1}\,{\rm{subject}}\,{\rm{to}}\,{\bf{Y}}=O\cdot {\bf{x}}\mathrm{(0)},$$

where $\parallel {\bf{x}}\mathrm{(0)}{\parallel }_{1}={\sum }_{i\mathrm{=1}}^{N}|{{\bf{x}}}_{i}\mathrm{(0)|}$ is the *L*_1_ norm of **x**(0), while ${\bf{Y}}\in {{\mathbb{R}}}^{qM}$, and $O\in {{\mathbb{R}}}^{qM\times N}$. Here *M* is the number of continuous measurements made by messengers. Because of the linear independence of the rows in matrix *O* and the sparsity of **x**(0), it is feasible to reconstruct **x**(0) as *M* is much smaller than *T* + 1. We define *n*_*M*_ ≡ *M*/(*T* + 1) to compare with the data amount *T* + 1 required by conventional solution to **x**(0). To be more realistic, we include both measurement noise and uncertainties in the link weights in Eq. (2), which is reformulated as

11 $$\{\frac{{\bf{x}}(t)=\hat{A}(t){\bf{x}}(t-\mathrm{1),}}{\hat{{\bf{y}}}(t)=C{\bf{x}}(t)\cdot ({\bf{1}}+\varepsilon ),}$$

where the measurement **y**(*t*) is contaminated by white truncated Gaussian noise of zero mean and variance *σ*^2^: $\varepsilon \sim {\mathscr{N}}({\bf{0}},{\sigma }^{2}{\bf{1}})$, where ${\bf{0}}\in {{\mathbb{R}}}^{q}$ is zero vector and ${\bf{1}}\in {{\mathbb{R}}}^{q}$ is the one vector. We assume that the uncertainties in the link weights *W* are also truncated Gaussian: ${\hat{w}}_{ij}(t)={w}_{ij}(t\mathrm{)(1}+\varepsilon ^{\prime} )$, where $\varepsilon ^{\prime} \sim {\mathscr{N}}\mathrm{(0},\sigma {^{\prime} }^{2})$. The random noise is restricted to positive values to make sure that the values of measurements and link weights are nonnegative. Here we use multiplicative noise to ensure that, on average, the ratio of the measurements remains the same with or without noise during the dynamics. To quantify the performance of source localization, we use the standard AUROC (area under a receiver operating characteristic) metric^37^, where *AUROC* = 1 indicates the existence of a threshold to fully distinguish between sources and passive nodes whereas AUROC = 0.5 indicates that the two types of nodes cannot be distinguished (Sec. S7 in SI).

**Figure 5:**
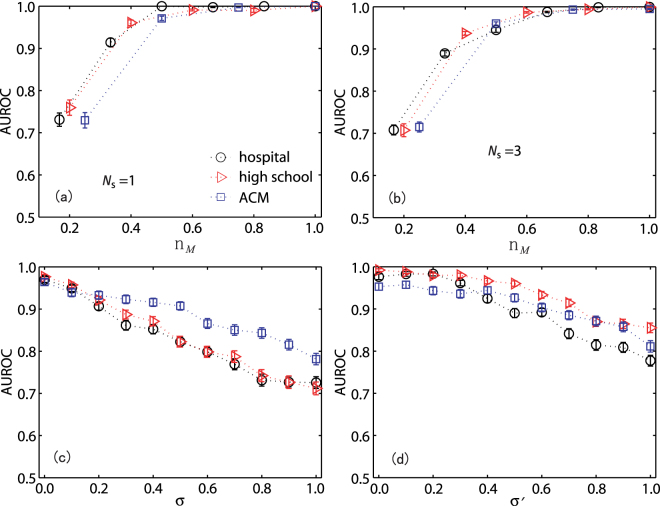
Performance of source localization for empirical networks. (**a**,**b**) AUROC as a function of *n*_*M*_ without noise for a single source (*N*_s_ = 1) and three sources (*N*_s_ = 3), respectively. (**c**,**d**) AUROC versus the measurement noise standard deviation *σ* and link weight standard deviation *σ*′, respectively. Parameters are *p* = 0.15 and *β* = 0.05. In (c,d), the values of *n*_*M*_ are 0.5, 0.6 and 0.5 for hospital, high school and ACM, respectively. The time window is a day and there is a single source. All results are obtained by averaging over 500 independent realizations and the vertical bars indicate the standard error.

We use empirical networks (as in Fig. 4) to test the performance of our CS based source localization method. As shown in Fig. 5(a) and (b), AUROC increases with *n*_*M*_. When *n*_*M*_ is small, AUROC shows large deviation indicating that the location of sources largely affects the accuracy of source localization for given selected messengers; once *n*_*M*_ exceeds some value, say 0.5, AUROC is close to 1 and the standard deviation reduces a lot implying that all sources at any locations can be accurately located. We also compared the performance of source localization for different messenger selection strategies (See Sec. S4 and Fig. S5 in SI). Figure 5(c) and (d) show the localization accuracy versus measurement noise *σ* and weight uncertainty *σ*′. We see that relatively high accuracy can still be achieved even when the noise variance approaches unity. Nonetheless, in some simulations the AUROC is small (See Sec. S5 and Fig. S6 in SI for the distributions of AUROC) and we may improve these performances by increasing the number of messengers or the length of observation time. Further efforts are still needed to see how to balance the cost of adding more messengers or increasing observation time.

**Figure 6:**
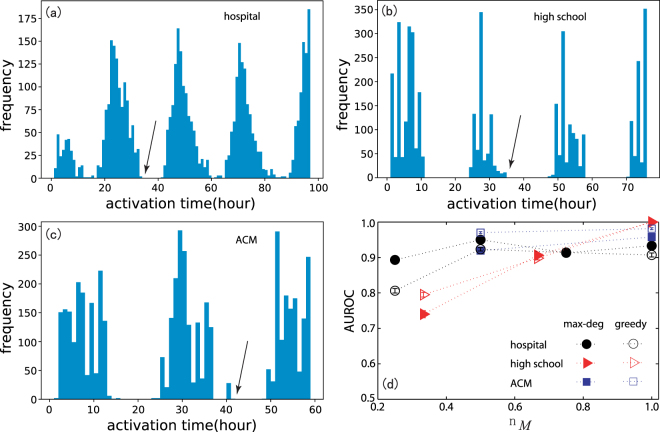
(**a**–**c**) Activation time distributions for empirical networks. The dividing point of the dataset is shown in black arrow. (**d**) Localization performance as a function of *n*_*M*_ for three sources (*N*_s_ = 3) without noise for greedy algorithm and max-deg strategy. *β* = 0.05. The results are obtained by averaging over 500 independent realizations and the vertical bars indicate the standard error. Here we use the first part of the data to choose messengers and locate the sources on the networks constructed with the second part. The time window is a day.

In real systems, we cannot know the time-varying network structure in advance, which prevents us from selecting the optimal messengers. However, if the network structure evolves with periodicity or follows some patterns, e.g., the activation dynamic of each edge remains stable for a long period, we can construct a rough network based on the past interactions and select messengers using its structural properties, e.g., nodal degree and estimated observable range. To test the effectiveness of our method under such situation, we divide the time-varying network into two parts according to the order of each edge’s activation time: the first part with which a rough network is constructed and a set of messengers is selected, and the second part within which the source localization is applied. Figure 6(a–c) display the activation time distributions of the three empirical networks, which indicates circadian rhythms, and illustrate the dividing time point used in the simulation. Messengers are selected using greedy algorithm and max-deg strategy ensuring full observable of the first part network, and are further used to locate the sources on the second part network. As shown in Fig. 6(d), our sources localization method shows a good performance for both strategies on the empirical networks.

## Discussions

Source localization is significant for preventing negative diffusion processes and reducing damages. Combining structural observability theory with sparse signal reconstruction, we succeed in developing a general framework for locating multiple diffusion sources in time varying networks, an extremely challenging problem in complex dynamical systems. The framework allows us to define an observable centrality for each node and to locate any number of sources by observing a small number of messenger nodes with larger values of observable centrality and exploiting the natural sparsity of sources. Appealing features of our framework include requirement of only small amounts of measurements and robustness against noise and uncertainties in system parameters. We offer analytic formulas for the observable centrality and the minimum number of messenger nodes, which are validated using model and empirical networks. A general finding based on our framework is that large degree nodes produce more valuable information than small degree nodes, an opposite result to that for static networks based on structural observability theory. As a result, choosing larger degree nodes as messenger nodes is more efficient to locate multiple sources in time varying nodes; in contrast, small degree nodes are often selected as messenger nodes in static networks. A counterintuitive finding is that sources in a more rapid varying network can be located more readily than in a slowly changed network. A heuristic explanation for this phenomenon is that frequent changes of the network structure in general produce more independent path in the static mapping of the original time varying network. As a result, the number of necessary messenger nodes is reduced and the sources become relatively easier to be localized. When dealing with time-varying networks, forward-planing problem is an unavoidable issue, because in many real systems the future structure of the time-varying network cannot be obtained in advance. While if the network structure evolves periodically or following some patterns, we can select messengers by fully exploiting the structural information embedded in the past interactions; If the evolution of time-varying network is totally random, then selecting messengers randomly may be the only way. In this paper, multiplicative noise is considered to test the robustness of our method, although the average performance is still satisfied, the worst cases are even worse than that of random guess (AUROC < 0.5) when the noise is strong. Therefore, it is very important to develop a more robust and efficient inference framework that can deal with different noise settings. One possible improvement is relaxing the object function *Y* = *O* ⋅ *X* to ||*Y* − *O* ⋅ *X*(0)||_2_ + *λ*||*X*(0)||_1_ in the cost of adding a tuning parameter *λ*. Another possible way is to develop a probabilistic approach which can utilize the distribution of noise to give a maximum likelihood estimation of the sources.

Our framework has potential applications in addressing many problems relevant to source localization, such as consensus, synchronization on power grid networks, locating the sources of epidemic spreading and rumor spreading in society, online social communities and computer networks. Moreover, our work has implications in disease diagnosis and therapy, such as identify focus sources of epilepsy and tumors in human body. Because of the significance and broad application potential of the source localization problem, we expect that the theory and practical algorithms presented in this work will stimulate further efforts, e.g., a more efficient and accurate algorithm to identify a minimum set of messenger nodes and a new framework available for systems with strong nonlinear properties.

### Data availability statement

Data can be accessed at http://www.sociopatterns.org/datasets.

## Electronic supplementary material

Supplementary Information
