# Supplementary material for: Locating multiple diffusion sources in time varying networks from sparse observations
Source: Sci Rep. 2018 Feb 8;8:2685. doi: 10.1038/s41598-018-20033-9 (PMC5805710; doi:10.1038/s41598-018-20033-9)
Supplement: Supplementary file 1 — Supplementary Information [file 41598_2018_20033_MOESM1_ESM.pdf]

Supplemental Information for

Locating multiple diffusion sources in time varying networks from sparse observations

Zhao-Long Hu, Zhesi Shen, Shinan Cao, Boris Podobnik, Huijie Yang, Wen-Xu Wang, and Ying-Cheng Lai

S1. Locating multiple sources in continuous time dynamical networks

In the main text we focus on discrete time varying systems. Here, we show that our source locating framework can readily be extended to continuous time dynamical networks. For concreteness, we consider the following network diffusion model:

$$\dot{x}_i(t) = \beta \sum_{j=1}^{N} \left[w_{ij}(t) x_j(t) - w_{ji}(t) x_i(t) \right], \tag{S1}$$

where $x_i(t)$ is the state of node i at the time t, β is the diffusion coefficient (constant), and $w_{ij}(t)$ [$w_{ji}(t)$] is the weight of the directed link from node j to node i (i to j) at time t. Combining Eq. (S1) and outputs from these nodes, we have

$$\begin{cases} \dot{\mathbf{x}}(t) = \beta L(t)\mathbf{x}(t), \\ \mathbf{y}(t) = C\mathbf{x}(t), \end{cases} \tag{S2}$$

where $\mathbf{x}(t) \in \mathbb{R}^N$ represents the complete state of the network system at time t, N is the number of nodes. The matrix $L(t) = W(t) - D(t)$ with $W(t) \in \mathbb{R}^{N\times N}$ is the adjacency matrix of elements $w_{ij}(t)$, $D(t) \in \mathbb{R}^{N\times N}$ is a diagonal matrix with element $d_i(t)$ representing the total out-weight $\sum_{j\in\Gamma_i(t)} w_{ji}(t)$ of node i, $\Gamma_i(t)$ is the set of neighbors of node i at time t, $\mathbf{y}$(t) is the vector of q outputs at time t, and $C \in \mathbb{R}^{q\times N}$ is the output matrix with q denoting the number of messenger nodes. The output response of the system is

$$\mathbf{y}(t) = C\Phi(t, t_0)\mathbf{x}(t_0), \tag{S3}$$

where $\Phi(t, t_0) = e^{\int_{t_0}^{t} \beta L(\tau) d\tau}$ is the state transition matrix, which can be computed from the adjacency matrix $W(t)$. For convenience, we stack all the outputs $\mathbf{y}(t)$ into a vector: $\mathbf{Y} = [\mathbf{y}(t_0); \cdots; \mathbf{y}(t_0 + 0.1); \cdots; \mathbf{y}(t_0 + 0.2); \cdots; \mathbf{y}(t_0 + t)]$. Intuitively, N snapshot measurements of the network state are needed for a unique solution. Without loss of generality, we sample at

time interval t_T to obtain

$$\begin{pmatrix} \mathbf{y}(t_0) \\ \mathbf{y}(t_0+t_T) \\ \vdots \\ \mathbf{y}(t_0+(N-1)t_T) \end{pmatrix} = \begin{pmatrix} C \\ C\Phi(t_T, t_0) \\ \vdots \\ C\Phi((N-1)t_T, t_0) \end{pmatrix} \mathbf{x}(t_0) = O \cdot \mathbf{x}(t_0), \qquad \text{(S4)}$$

where the matrix $O \in \mathbb{R}^{qN \times N}$ is the observability matrix in canonical control theory. A unique solution of Eq. (S4) exists and the state vector $\mathbf{x}(t_0)$ at initial time is observable if and only if the rank condition $\mathrm{rank}(O) = N$ is satisfied. These considerations establish the applicability of our framework of sources localization to continuous time dynamical network systems.

S2. Structural observability of time varying networks

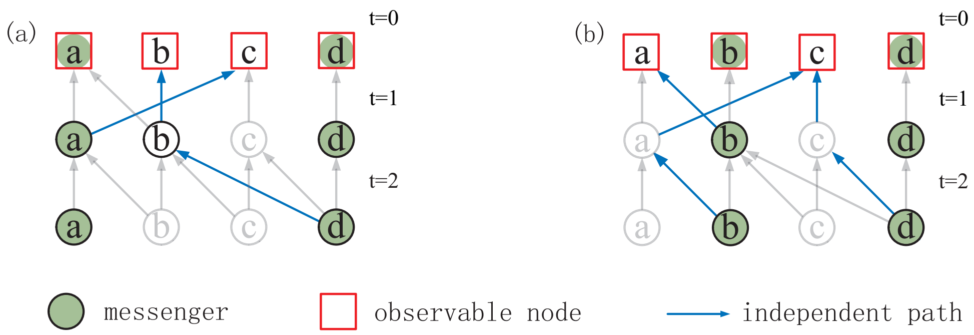

Fig. S1. Independent paths and identification of all minimum sets of messenger nodes in a time varying network. (a) Two independent paths for observing nodes a and d. The network is fully observable because we have $N_{\mathrm{OR}}(\{a, d\}) = 4$. (b) Two independent paths for observing nodes b and d. The network is also fully observable. The blue arrow lines specify independent paths from messenger to other nodes. The sources can be located from the messenger set $\{a, d\}$ or $\{b, d\}$, i.e., the configuration of the minimum messengers that ensure full localization of the diffusion sources may not be unique.

1. Identifying all minimum sets of messenger nodes in a time varying network

Figures S1(a,b) show, for the time varying network described in Fig. 1 in the main text, two configurations of the minimum messenger set that guarantees full observability of the network. For example, as shown in Fig. S1(a), when a and d are messengers, there are two independent paths to other nodes (node b and c), and we have $N_{\mathrm{OR}}(\{a, d\}) = 4$, entailing that the network is fully observable. While the minimum number of messengers is two, the configuration of the messengers is not unique: often there are more than one configuration of messenger node set that can ensure full observability of the network. For example, if we choose nodes b and d to be

messengers, the network is also fully observable. The independent paths in this case are shown in Fig. S1(b).

2. Relationship between independent paths and the number of distinct activations

We present examples to demonstrate that the number of independent paths starting from a messenger node is the number of distinct activations of the links going out from this node. Note that, at a certain activation time t, there is at most one independent path starting from a messenger node to the top layer ($t = 0$). For example, as shown in Fig. S1(a), if we choose node d as a messenger node, there are two paths starting at $t = 2$, i.e., $d \to b$ and $d \to c$. At $t = 1$, node b also has two paths, i.e., $b \to a$ and $b \to b$. Then for messenger d, there are three paths, i.e., $d \to b \to a$, $d \to b \to b$ and $d \to c \to c$. However, it is necessary to choose one of them to be an independent path, because two of them are dependent paths. Since d has only one activation time, i.e., $t = 2$, there is a single independent path from d.

As another example, if we choose b to be a messenger node, as shown in Fig. S1(b), it has two different activation times: $t = 1$ and $t = 2$. As a result, there are two independent paths from b: $b \to a \to c$ starting from $t = 2$ and $b \to a$ starting from time $t = 1$. This example also indicates that the number of independent paths from b is at most the number of distinct activations. It can happen that two independent paths starting from two different activation times share the same ending node, rending the number of independent paths less than distinct activation times. However, such a situation is rare if the network does not possess a tree structure, as there usually exist many independent paths that do not share an ending node.

The examples illustrate that $N_{\mathrm{OR}}(\{v\})$ can be approximated by $n_{\mathrm{OR}}(\{v\}) \approx (l_v + 1)/N$, where l_v is the number of different activations of node v, and the unity stems from the messenger node itself.

3. Derivation of the probability of having exactly l distinct activations

In the main text, we give the probability of having exactly l distinct activations for one node at activation time $z^1, \ldots, z^k$ on each edge. To derive it, we resort to the solution of the problem of partitioning a set of n objects into k non-empty subsets, in which the number of ways is the Stirling number of the second kind. The number of ways of assigning l different time tags to the total $z^1 + \cdots + z^k$ activation time is

$$\binom{T}{l} l! \times \frac{1}{l!} \sum_{j=0}^{l} (-1)^{l-j} \binom{l}{j} j^{z^1 + \cdots + z^k}, \tag{S5}$$

where the first part on the left of $\times$ is the ways of choosing l different time tags from T, and the right part is the Stirling number. Because of the restriction that, for each edge, its z activation times must be different, the above equation needs to be modified by replacing $j^{z^1+\cdots+z^k}$ with

$\prod_i^k \binom{j}{z^i}$ and the summation should start from $\max(z^1, \ldots, z^k)$. The final form is

$$\binom{T}{l} \sum_{j=\max(z^1,\ldots,z^k)}^{l} (-1)^{l-j} \binom{l}{j} \prod_i^k \binom{j}{z^i}. \tag{S6}$$

S3. Observable range associated with different messenger finding strategies

In this section, we provide a greedy optimization algorithm to find an approximately minimum set of messengers to achieve full observability of time varying networks. We also compare the performance of the greedy optimization with those of other degree-based messenger selection algorithms.

1. Greedy Optimization Algorithm

Our goal is to solve the following optimization problem

$$\max_{Q \subseteq V} R(Q), \tag{S7}$$

where Q is the set of selected messengers and $R(Q) \equiv \text{rank}[O(Q)]$ is the generic rank of O. The observability function $R(Q)$ has several properties, which can be used to speed up the greedy optimization algorithm. Firstly, we have $R(\emptyset) = 0$, i.e., nothing can be observed if we do not place any messenger. Secondly, $R(*)$ is nondecreasing, i.e., $R(Q_1) \leq R(Q_2)$ for all $Q_1 \subseteq Q_2 \subseteq V$. The third and most important property is the submodular property, i.e., for all placements $Q_1 \subseteq Q_2 \subseteq V$ and messenger $v \in V \setminus Q_2$, the following holds:

$$R(Q_1 \cup \{v\}) - R(Q_1) \geq R(Q_2 \cup \{v\}) - R(Q_2). \tag{S8}$$

Maximizing submodular functions in general is NP-hard. A commonly used optimization strategy is the greedy algorithm, which starts from the empty messenger set $Q_0 = \emptyset$ and iteratively, in step s, add the node v so as to maximize the marginal gain

$$\delta_v = \text{argmax}_{v \in V \setminus Q_{s-1}} R(Q_{s-1} \cup \{v\}) - R(Q). \tag{S9}$$

The algorithm stops once full observability is achieved. While evaluating the observability function $R(Q)$ based on the maximum flux carries the computational complexity $O(N|E|)$ and is therefore demanding, what is needed is an approximation evaluation of qN functions if we select q messengers, where $|E|$ is the number of edges of the network. We can exploit the property of submodularity further to reduce the function evaluations. In particular, we propose the following improved greedy optimization algorithm.

1. Calculate the observability centrality of every node and obtain a list in a descending order. The observability centrality can be approximated by the number l of distinct activations.

2. For $Q = \emptyset$, calculate the increment δ_v for each $v \in V$, take the nodes in the descending order of δ_v, and add the node with the largest value of δ_v into Q.

3. Recalculate the δ_v for the top ranked node in the list $V \setminus Q$, and insert the node into the existing queue based on its marginal gain δ_v.

4. If the top ranked node remains in its top position, go to 5; else go to 3.

5. Add the top ranked node into Q and go to 3 until full observability is achieved.

The computational cost associated with the improved greedy algorithm and the original one is presented in Fig. S2. The improved algorithm shows a great reduction in the number of required iterations of computing marginal gain.

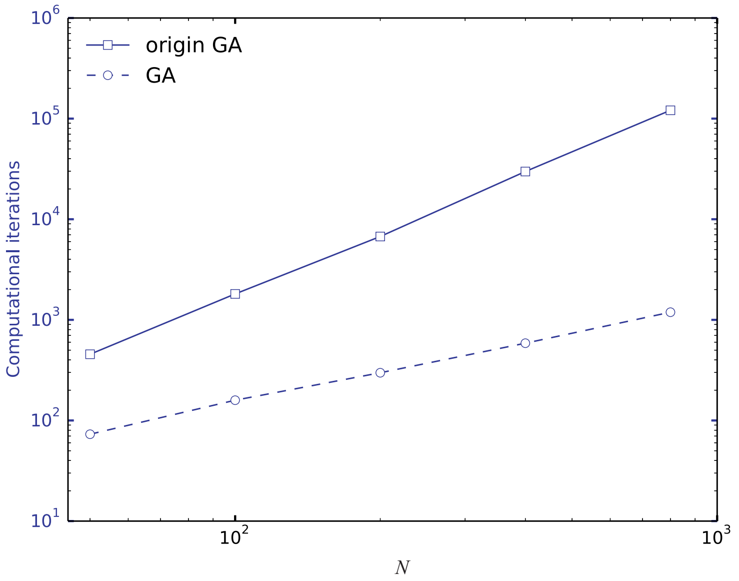

Fig. S2. Computational iterations of evaluating marginal gains for improved and original greedy algorithms. The square (solid and empty) points are for the original algorithm, and the circle (solid and empty) points are for the improved algorithm.

2. Observable range of model and empirical networks

To appreciate the performance robustness of the greedy algorithm, we study the following degree-based messenger selection strategies for comparison:

- $\max - \deg$: choose nodes with the largest degree,
- $\min - \deg$: choose nodes with the smallest degree,
- $\mathrm{ran} - \deg$: choose nodes at random.

We first compute the theoretical result of $\max-\deg$ based on the descending order of degree: $k = N-1, N-2, \cdots, 1, 0$ with degree distribution $p(k)$. A fraction q/N of messenger nodes are chosen from this list, and the normalized observable range is

$$n_{\rm OR}(Q) = \sum_{i\in Q} p(k_i) N_{\rm OR}(k_i), \tag{S10}$$

where $N_{\rm OR}(k_i)$ is the observable centrality for the messenger i with degree k_i.

For ER networks, $p(k)$ is approximately $e^{-\langle k\rangle}\langle k\rangle^k/k!$. For SF networks, in the thermodynamic limit, we have $p(k) = 2(\langle k\rangle/2)^2 k^{-3}$. For the number q of messenger nodes, we combine the form of $p(k)$, $\sum_{k^*}^{N-1} p(k) \leq q/N$, and $\sum_{k^*-1}^{N-1} p(k) \geq q/N$ to determine the degree threshold k^*. Let $q/N = \sum_{k^*}^{N-1} p(k) + \Delta$. We have $\Delta = q/N - \sum_{k^*}^{N-1} p(k)$ and obtain $n_{\rm OR}(Q)$ as

$$n_{\rm OR}(Q) = [\sum_{k^*}^{N-1} n_{\rm OR}(k) p(k) N] + \Delta n_{\rm OR}(k^*-1). \tag{S11}$$

The normalized observable centrality of node of degree k is shown in the main text, i.e.,

$$n_{\rm OR}(k) = (\langle l\rangle + 1)/N, \tag{S12}$$

where $\langle l\rangle$ representing the distinct activations of node with degree k is given in the main text. Combining Eqs. (S11) with (S12), we can obtain the normalized observable range of messenger set Q with the largest degree.

For the strategy $\min-\deg$, we treat it in a similar way. While for the strategy $\mathrm{ran}-\deg$, it is only necessary to compute $\langle n_{\rm OR}\rangle q$, where $\langle n_{\rm OR}\rangle = \sum_k p(k) n_{\rm OR}(k)$.

We analyze the behavior of $n_{\rm OR}$ with different messenger selection strategies. Figure S3 shows that $n_{\rm OR}$ from the max-deg strategy is quite close to that from the greedy strategy, e-specially for relatively larger values of $z_{\rm max}$, suggesting the local information based max-deg strategy as an efficient alternative to the greedy strategy that is based on global optimization. Another finding is that a small fraction p of messenger nodes is sufficient to fully locate multiple sources for both ER and SF networks. In addition, the value of $n_{\rm OR}$ with the min-deg strategy is the smallest for both ER and SF networks, a result distinct from that for static networks. The lattice networks are used here for comparison.

We also test our framework using three empirical time-dependent networks (Table S1). The results in Fig. S4 show that only a quite small value of p is needed to ensure full localization of diffusion sources in the empirical networks. Similar to ER and SF networks, the resulting $n_{\rm OR}$ value from the min-deg strategy is the smallest for all the empirical networks.

For both model and empirical networks, numerical calculations are in good agreement with theoretical predictions, especially for larger values of $z_{\rm max}$. Naively, one might expect that sources in a more frequently changing network would be more difficult to be identified. However, we find that in both model and real networks, diffusion sources in more rapidly changing networks can be located more readily (cf., the case with $z_{\rm max} = 1$ versus that with $z_{\rm max} = 5$ in Fig. S3 and hour versus day in Fig. S4).

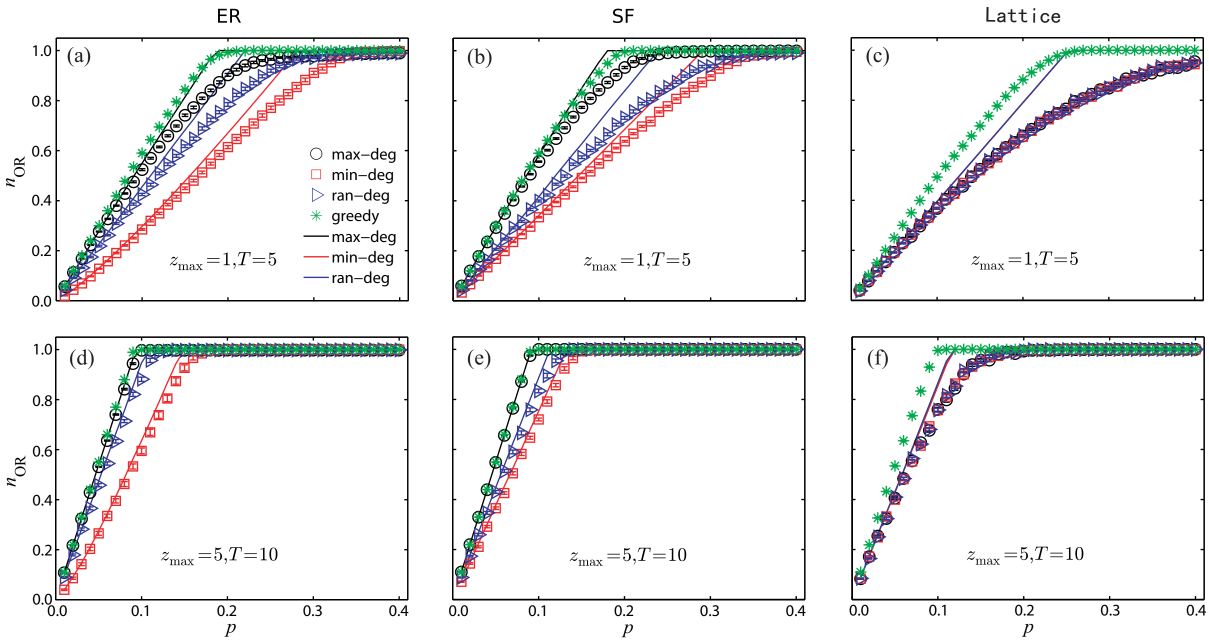

Fig. S3. Normalized observable range for different types of networks. In ER, SF and lattice networks, for different values of T and $z_{\max}$, the normalized observable range n_{OR} as a function of the fraction p of messengers for four strategies: $\max - \mathrm{deg}$, $\min - \mathrm{deg}$, $\mathrm{ran} - \mathrm{deg}$, and greedy for (a-c) $z_{\max} = 1$ and $T = 5$, (d-f) $z_{\max} = 5$ and $T = 10$, where (a,d), (b,e) and (c,f) are for ER, SF, and lattice networks, respectively. The different symbols indicate the corresponding simulation results. The theoretical predictions denoted by the solid curves for strategies $\max - \mathrm{deg}$, $\min - \mathrm{deg}$, $\mathrm{ran} - \mathrm{deg}$, are from Eq. (3) in the main text. The networks size is $N = 100$ and the average degree is $\langle k \rangle = 6$ for ER and SF networks, and $\langle k \rangle = 4$ for lattice networks. All results are obtained by averaging over 50 independent realizations and the vertical bars indicate the standard error.

S4. Sources localization of different messenger selection s-trategies

With the above definition of strategies for choosing messengers, here we study the performance of source localization for different messenger selection strategies. As shown in Fig. S5, the performance of max-deg strategy and greedy strategy are quite similar, and it is slightly better than that of ran-deg strategy. Meanwhile, the min-deg strategy perform the worst due to the lowest observing range given the same number of messengers compared to other strategies.

S5. Distribution of AUROC

In this section, we study the distribution of the value of AUROC for sources localization with different noise amplitudes. From Fig. S6(a-c), we can find that when there is no noise, $\sigma = 0$, all the values of P(AUROC=1) exceed 0.85. For hospital and high school data set-s, P(AUROC=1)≈ 0.95. The value P(AUROC$= 0.5$) for ACM is about 0.05, suggesting that

there is about 0.05 chance as the case of random guess. Fig. S6(d-f) show that there may exist AUROC< 0.5, but the probability is low even under a strong noise environment. For instance $\sigma = 1$, F(AUROC=0.5)< 0.12. For ACM data set, noise doesn't seem to affect the outcome. As a whole, Fig. S6 indicates that the probability to find a large AUROC is high, and the rest of AUROC almost obey an uniform distribution.

S6. Description of empirical networks

We use three empirical time varying networks: Hospital, High school and ACM. All the three data sets represent active contacts during 20-second intervals of data collection. The characteristics of the three empirical networks are listed in Table S1. For simplicity, we use an hour or a day as the time window.

S7. Performance assessment of sources localization

The area under a receiver operating characteristic (AUROC) is a widely used statistical characteristic in engineering, medicine, and physics, which measures the area under a probability curve that ranks a randomly chosen positive event higher than a randomly chosen negative one. AUROC for source localization is defined in terms of true positive rate (TPR) and false positive rate (FPR), defined as

$$\mathrm{TPR}(s) = \frac{\mathrm{TP}(s)}{P} \quad \text{and} \quad \mathrm{FPR}(s) = \frac{\mathrm{FP}(s)}{N-P}, \tag{S13}$$

where s is the cutoff (threshold) in the list of the reconstructed state $x_i(t)$ at time t, $\mathrm{TP}(s)$ $[\mathrm{FP}(s)]$ is the number of true (false) positives in the top s reconstructed values of $x_i(t)$, and P ($N - P$) is the number of positives (negatives) in the gold standard. AUROC is the area under the TPR-FPR curve.

Table S1. Characteristics of the three empirical time-dependent networks. The quantities N and $|E|$ denote the network size and the number of contacts, respectively. Al-l three networks are undirected. The structural data of the networks are available at: http://www.sociopatterns.org/datasets/.

Data sets Name	N	$\|E\|$	Duration	Description
Hospital	75	32424	97 hours	network of contacts between patients, patients and health-care workers (HCWs) and among HCWs in a hospital ward in Lyon, France, December 6, 2010. The study included 46 HCWs and 29 patients
High school	126	28561	76 hours	network of contacts between students of three classes in a high school in Dec. 2011, in Marseilles France
ACM	113	20818	59 hours	the ACM Hypertext 2009 conference: the dynamical network of face-to-face proximity of 110 conference attendees

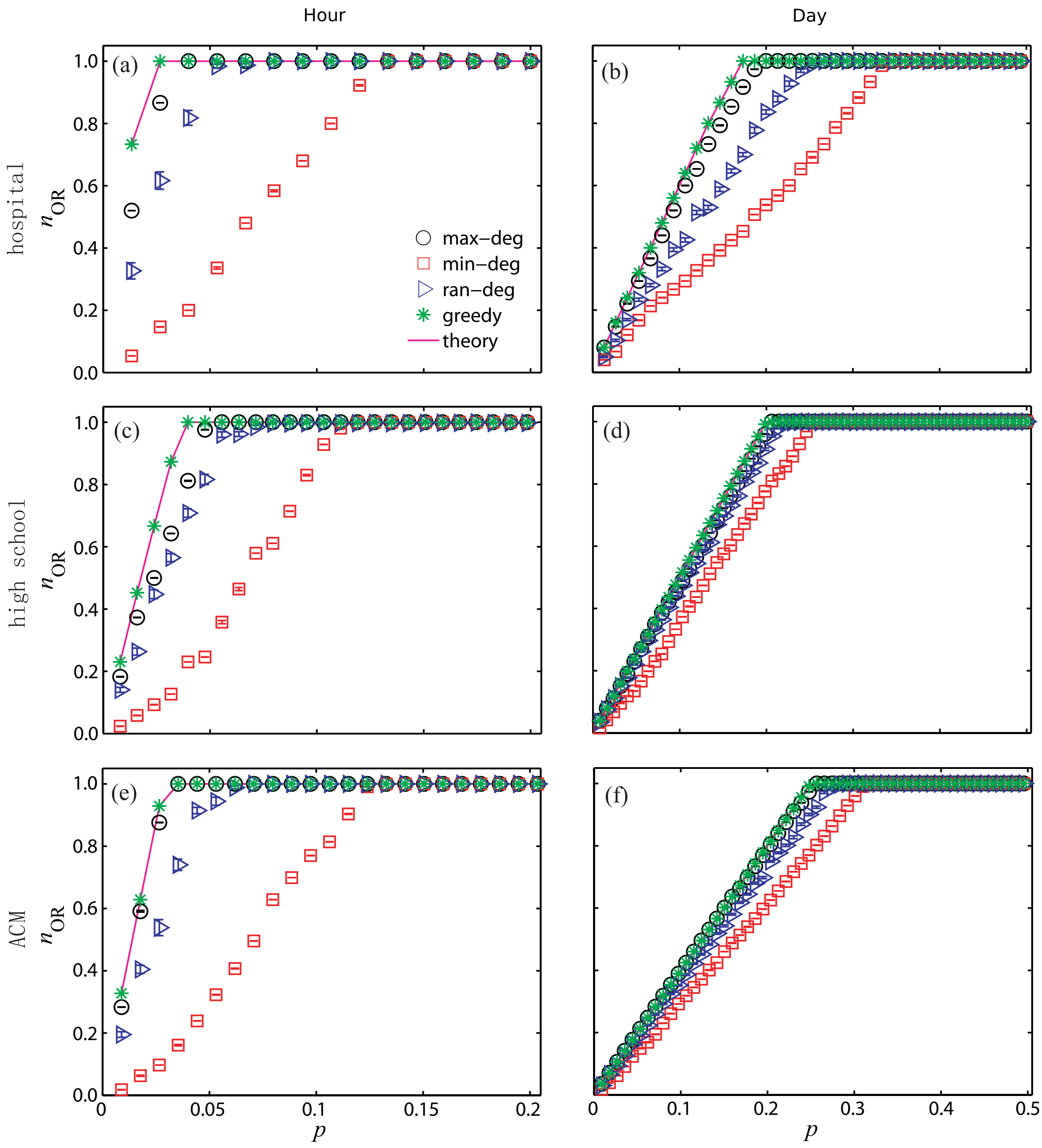

Fig. S4. Observable range for empirical time varying networks. Normalized observable range $n_{\rm OR}$ as a function of the fraction p of messengers for three empirical time varying networks associated with four strategies: $\max-\deg$, $\min-\deg$, $\mathrm{ran}-\deg$ and greedy: (a,b) hospital network, (c,d) high School network, and (e,f) ACM. For panels (a,c,e), the time window is an hour. For panels (b,d,f), the time window is a day. The theoretical predictions denoted by solid curves are from the formula $n_{\rm OR}(Q) \approx \sum_{i\in Q}(l_i+1)/N$ in the main text. More details of the empirical networks and the meaning of time window are described in Table S1. All results are obtained by averaging over 50 independent realizations and the vertical bars indicate the standard error.

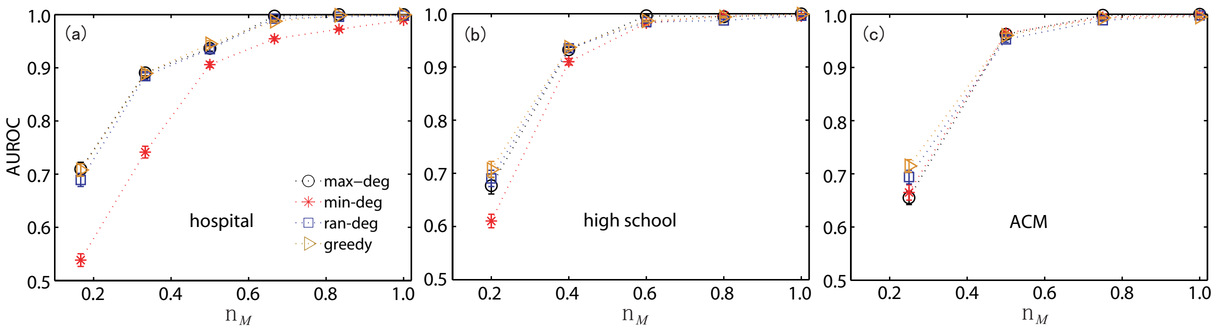

Fig. S5. Performance of source localization for different messenger selection strategies. AUROC as a function of n_M without noise for hospital (a), high school (b) and ACM (c), respectively. Parameters are $p = 0.15$, $\beta = 0.05$ and $N_s = 3$. The time window is a day. All symbols are obtained by averaging over 500 independent realizations with the vertical bars indicating the standard error.

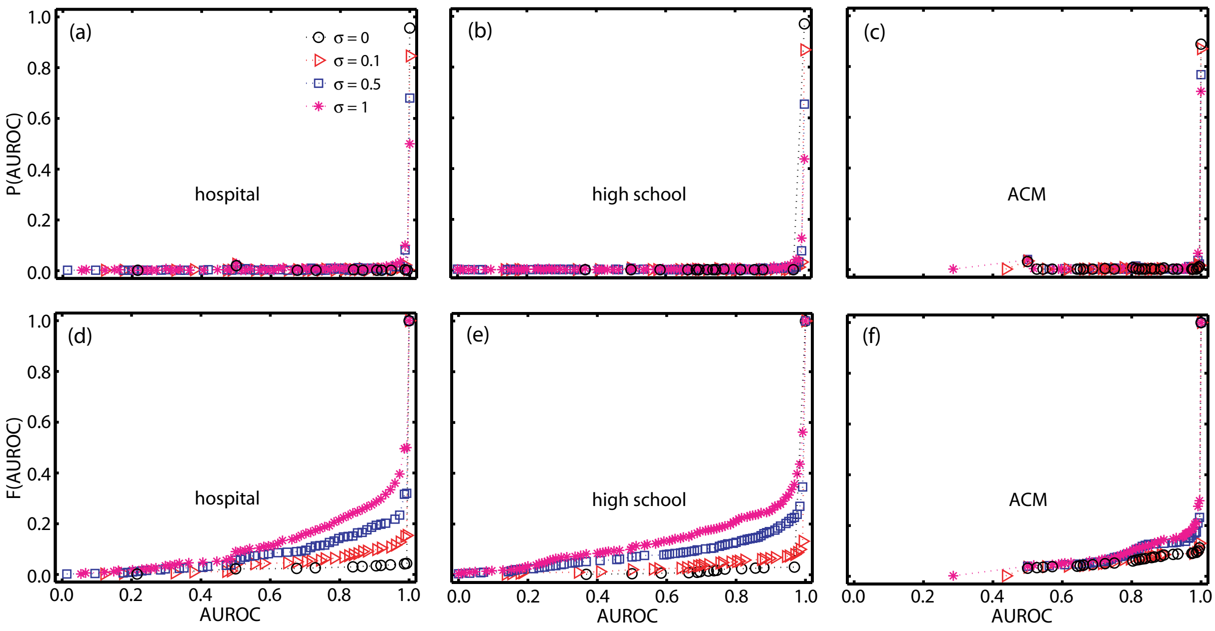

Fig. S6. Distribution of AUROC for different noise amplitudes. Distribution of AUROC P(AUROC) for hospital (a), high school (b) and ACM (c), respectively. Cumulative distribution of AUROC F(AUROC) for hospital (d), high school (e) and ACM (f), respectively. The standard deviation of measurements σ is set to 0, 0.1 0.5 and 1. Others parameters are $p = 0.15$, $\beta = 0.05$ and $N_s = 1$. The time window is a day. The distribution results are obtained by 500 independent realizations.
